# GPN-1/glypican and UNC-52/perlecan do not appear to function in BMP signaling to pattern the *C. elegans* postembryonic mesoderm

**DOI:** 10.17912/micropub.biology.000437

**Published:** 2021-08-13

**Authors:** Melisa S DeGroot, Robert Greer, Jun Liu

**Affiliations:** 1 Department of Molecular Biology and Genetics, Cornell University, Ithaca, NY. USA

## Abstract

Heparan sulfate proteoglycans (HSPGs) are diverse macromolecules consisting of a protein core modified with glycosaminoglycan (GAG) chains. HSPGs, including glypicans and perlecans, have been implicated in shaping the extracellular matrix (ECM) to affect growth factor signaling. Here, we tested if GPN-1/glypican**or UNC-52/perlecan plays a role in the bone morphogenetic protein (BMP) signaling pathway in patterning the *C. elegans *postembryonic mesoderm. Using the suppression of* sma-9(0) *(Susm)**assay, we found that animals carrying mutant alleles of *gpn-1* or *unc-52* do not exhibit any Susm phenotype. We also tested and found that the two glypicans GPN-1 and LON-2 do not share functional redundancy in the BMP pathway. Our results suggest that GPN-1/glypican and UNC-52/perlecan do not play a major role in the *C. elegans* BMP pathway, at least in patterning of the postembryonic mesoderm.

**Figure 1. The Susm phenotypes of lon-2, gpn-1 and unc-52 mutants:**
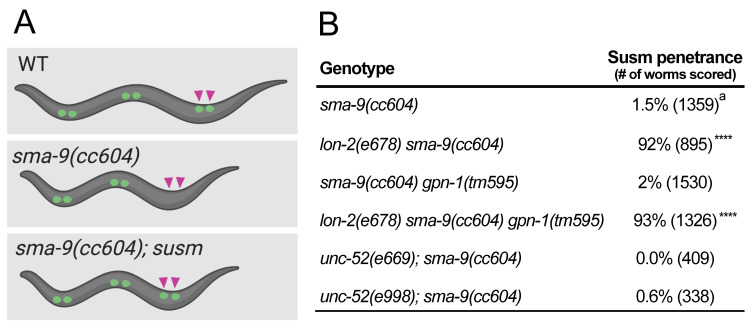
A) Diagram depicting the coelomocyte (CC) phenotype of wildtype (WT), *sma-9(cc604)*, or *sma-9(cc604); susm* worms. *sma-9(cc604)* mutants lack the two M-derived CCs located in the posterior of WT or *sma-9(cc604); susm* worms (purple arrows). Created using BioRender.com (version 2021). B) Table showing the penetrance of the Susm phenotype of double mutant strains between *sma-9(cc604)* and mutant alleles of the specified genes (thanks to CGC). The Susm penetrance refers to the percent of animals with one or two M-derived CCs as scored using the CC::GFP reporter. For each genotype, two independent isolates were generated (as shown in the strain list), three to seven plates of worms from each isolate were scored for the Susm phenotype at 20°C, and the Susm data from the two isolates were combined and presented in the table. ^a^ The lack of M-derived CCs phenotype is not fully penetrant in *sma-9(cc604)* mutants. Statistical analysis was conducted by comparing the double or triple mutants with the *sma-9(cc604)* single mutants. **** *P*<0.0001 (unpaired two-tailed Student’s *t*-test). No significant difference was detected between *lon-2(e678) sma-9(cc604)* double mutants and *lon-2(e678) sma-9(cc604) gpn-1(tm595)* triple mutants.

## Description

Heparan sulfate proteoglycans (HSPGs) are macromolecules composed of a protein core decorated with glycosaminoglycan (GAG) chains. These highly diverse molecules are classified based on their localization at the membrane or being secreted as part of the extracellular matrix (ECM), such as glypicans or perlecans, respectively (Sarrazin *et al.* 2011). HSPGs have been shown to play a structural role in the ECM as well as in the distribution of growth factors, such as transforming growth factor-β (TGF-β), fibroblast growth factor (FGF), Wnt, and Hedgehog (Hh), within tissues through highly specific binding interactions (Lin and Perrimon 2000). In vitro studies have demonstrated that human perlecan can bind to the BMP2 ligand (Decarlo *et al.* 2012). Understanding the biological roles of HSPGs in cell signaling has implications in disease diagnosis and potential treatment.

Bone morphogenetic protein (BMP) belongs to the TGF-β superfamily of signaling molecules. In *C. elegans*, the BMP signaling pathway is known to regulate multiple processes, including body size and postembryonic mesoderm patterning (Savage-Dunn and Padgett 2017). We have shown that mutations in the BMP pathway exhibit the Susm phenotype, namely, they can specifically suppress the mesoderm defects of *sma-9(0)* mutants (Figure 1A, Foehr *et al.* 2006). We further showed that the Susm assay is a highly specific and sensitive assay for BMP signaling defects (Liu *et al.* 2015). Previous work has demonstrated that LON-2/glypican negatively regulates the BMP pathway, and it has been postulated that LON-2 functions by binding to and sequestering the ligand (Gumienny *et al.* 2007). We have found that the *lon-2(e678)* null mutants exhibit the Susm phenotype (Figure 1B; Foehr *et al.* 2006; Liu *et al.* 2015). Here we aim to determine if GPN-1/glypican or UNC-52/perlecan plays a role in BMP signaling by using the Susm assay.

*gpn-1* encodes a glypican, a membrane localized HSPG. *gpn-1* is expressed in the pharynx and ventral nerve cord (VNC) during embryogenesis (Hudson *et al.* 2006). Previous studies have shown that *gpn-1* mutants do not have a body size phenotype, and *gpn-1* cannot substitute for *lon-2*, which encodes another glypican, to regulate body size (Gumienny *et al.* 2007; Taneja-Bageshwar *et al.* 2013). However, the Drosophila glypicans Dally and Dally-like are known to function in the BMP pathway by regulating the distribution of the BMP ligand within the ECM (Belenkaya *et al.* 2004; Norman *et al.* 2016). Furthermore, the *C. elegans* BMP ligand, DBL-1, is secreted from cells of the VNC (Suzuki *et al.* 1999), the same tissue that expresses *gpn-1*. We sought to test if *gpn-1* plays a role in the BMP pathway by using the more sensitive Susm assay. We assayed for the Susm phenotype of *gpn-1(tm595)*, a deletion allele lacking part of exon 2 and all of exon 3 of the *gpn-1* gene. We found that *gpn-1(tm595)* animals exhibited a 2% penetrance of the Susm phenotype, within the range of background observed in the *sma-9(cc604)* single mutants (Foehr *et al.* 2006; Liu *et al.* 2015).

Previous studies have shown that *gpn-1* and *lon-2* act redundantly in hermaphrodite specific neuron migration (Kinnunen 2014). To test if these two glypicans may function redundantly in the BMP pathway, we examined the Susm phenotypes of *lon-2(e678) gpn-1(tm595)* double mutants. We found that *gpn-1(tm595)* does not enhance the penetrance of the Susm phenotype of *lon-2(e678)*. Taken together, these results suggest that *gpn-1* does not play a major role in the BMP pathway.

Perlecan is a secreted HSPG, characterized by its localization to the basement membrane. Human perlecan has been shown to be a key component of basement membranes that can bind and sequester growth factors, such as FGFs and VEGFs (Costell *et al.* 1999; Mongiat *et al.* 2000; Ishijima *et al.* 2012). While a study has demonstrated that TGF-β signaling regulates the expression of perlecan (Dodge *et al.* 1995), a role for perlecan in the TGF-β signaling pathway has not been shown. *C. elegans* has one homolog of the human perlecan: *unc-52* (Rogalski *et al.* 1993)*.* Localized to the basement membrane of body wall muscles (BWM), UNC-52 has been shown to play a critical role in the assembly and maintenance of the BWM myofilaments (Mullen *et al.* 1999). Interestingly, previous studies have shown that *unc-52* genetically interacts with *dbl-1*/BMP during distal tip cell (DTC) migration (Merz *et al.* 2003). We tested if *unc-52* plays a role in the BMP pathway by using the *sma-9* suppression assay. We used two nonsense alleles of *unc-52*, *e669* and *e998,* which introduce an early stop in exon 17 and 18, respectively. Both *e669* and *e998* were among the multiple *unc-52* alleles used in the study by Merz *et al.* (2003). Neither *unc-52(e669)* nor *unc-52(e998)* exhibited any Susm phenotype (Figure 1B). Although not all UNC-52 isoforms are abolished in these two mutants, our results suggest that UNC-52/perlecan is unlikely to play a major role in the BMP pathway in *C. elegans*.

In summary, using the highly specific and sensitive Susm assay, we have found that unlike LON-2/glypican, GPN-1/glypican and UNC-52/perlecan do not appear to play a major role in the *C. elegans* BMP pathway. The *C. elegans* genome contains three other HSPGs, SDN-1/syndecan, AGR-1/agrin, and CLE-1/collagen XVIII (*cle-1*). It remains to be determined whether any of them play a role in BMP signaling.

## Reagents

***Strains:***

**Table:** 

LW0040:	*arIs37[secreted CC::gfp] I; cup-5(ar465) III; sma-9(cc604) X*
LW5711:	*arIs37[secreted CC::gfp] I; unc-52(e669) II; cup-5(ar465) III; sma-9(cc604) X isolate 1*
LW5712:	*arIs37[secreted CC::gfp] I; unc-52(e669) II; cup-5(ar465) III; sma-9(cc604) X isolate 2*
LW5734:	*arIs37[secreted CC::gfp] I; unc-52(e998) II; cup-5(ar465) III; sma-9(cc604) X isolate 1*
LW5735:	*arIs37[secreted CC::gfp] I; unc-52(e998) II; cup-5(ar465) III; sma-9(cc604) X isolate 2*
LW5778:	*arIs37[secreted CC::gfp] I; cup-5(ar465) III; lon-2(e678) sma-9(cc604) X isolate 1*
LW5779:	*arIs37[secreted CC::gfp] I; cup-5(ar465) III; lon-2(e678) sma-9(cc604) X isolate 2*
LW5709:	*arIs37[secreted CC::gfp] I; cup-5(ar465) III; sma-9(cc604) gpn-1(tm595) X isolate 1*
LW5710:	*arIs37[secreted CC::gfp] I; cup-5(ar465) III; sma-9(cc604) gpn-1(tm595) X isolate 2*
LW5713:	*ccIs4438 [intrinsic CC:::gfp] III; ayIs2[egl-15p::gfp] IV; lon-2(e678) sma-9(cc604) gpn-1(tm595) X isolate 1*
LW5714:	*ccIs4438 [intrinsic CC:::gfp] III; lon-2(e678) sma-9(cc604) gpn-1(tm595) X isolate 2*

***Primers used for genotyping:***

***cc604*:** MLF-69: CGCAACAAGTTCATTCTCCA. MLF-70: CTTGGCTAAGATCCCATGCT. Sequence using LW-40: TCCGACTTGACACTTCATCAGC.

***e678*:** JKL-1053: TTGTATTGCTCTACCGGTCC. JKL-1054: TTGCCCGGAATTTCAACTGC. JKL-1055: TCAACTTACGGAAGCGATCG.

***e669*:** JKL-1871: TCCGTCAACTCTCTCGAAGG. JKL-1872: CACAAGTCGAAGCTCGTTAG. Sequence using JKL-1872.

***e998*:** JKL-1873: CAAAATCGTTAGTGGCCGAG. JKL-1874: CCTGTTCACTCCCACTTCTC. Sequence using JKL-1873.

***tm595*:** MSD-72: TGGCTTCACTGATTAGTACCGG. JKL-1868: ACCGTTCACATGGATCTTGAC. JKL-1870: CCATGCATACTCGCTGATCG.
